# Foreign Medico-Psychological Literature

**Published:** 1862-04

**Authors:** 


### FOREIGN MEDICO-PSYCHOLOGICAL LITERATURE.

Our Retrospect of current Foreign Medico-Psychological Literature will embrace the following subjects:

1. Report on a Case of Mental Derangement with Kleptomania.
2. Dr. Flemming on Insane Institutions and Insane Colonies.
3. Report of the Commission of the Medico-Psychological Society of Paris on Gheel.

1.—*Report on a Case of Mental Derangement with Kleptomania.* By Dr. Max Mauthner, Court Physician to the Provincial Court of Justice in Oedenburg, for the time being in Vienna.

Notice was given, upon the 12th June, 1860, to the District Court at Oedenberg, that the prisoner, post-office clerk, John P., had been suffering, from the 26th May to the 8th June, from violent inflammation of the brain accompanied by mania; and the conjecture was hazarded that, even during the time of his purloinings, he had not the full use of his understanding. Dr. Mauthner and the physician of the town were appointed to investigate the case.

(A) RESULT OF THE INQUIRY.

1. Prisoner, aged 19 years, born in Djejity, is of low stature, and spare appearance. 2. The form of the head regular; the oval, pale, youthful face beardless; eye large, moist, pupils dilated, the left larger than the right; the left corner of the mouth depressed on the right side. 3. Chest well formed, heart not enlarged; heart's action very rapid, first sound of the heart accompanied by a slight murmur; pulse in general weak, not full, 120; temperature of the skin rather low; skin of the hands generally blue, red; cool. 4. Sleeps little; is unrefreshed in consequence of frightful visions and harassing dreams. 5. Manner of expressing himself offers no particular abnormal features of the mind's activity; complains of singing and ringing in his ears. 6. He is tractable, good-humoured, sociable; his carriage and behaviour not in any way remarkable, except in his manner of salutation, which is somewhat childish. 7. Is the eldest son of a dresser of leather in Moravia, who possesses two establishments; was 10 years old when sent to school at Brünn; passed through both the under and upper school, and attended also for a year a commercial school. 8. After the examinations he entered, on the 21st Nov. 1858, into the service of the State in the capacity of a probationer at the District Finance Court at Brünn; after passing an examination on the 3rd April, 1859, he was sworn in as clerk; he was afterwards, on his own application, transferred to a similar situation in Oedenberg; and on the 29th Nov. of the same year he was released, by his own wish, from this latter situation, and entered the service of the Post-Office as clerk, where he remained until his incarceration for opening letters. 9. About fourteen days before his imprisonment he paid up his caution money. 10. During his studies and services as an unpaid clerk he had been sufficiently maintained by his parents, but had used his means with great economy, living much more sparingly than was necessary. 11. He says himself that he had great difficulty in learning, that his father often reproached him for his stupidity; says also that he always slept very little. 12. During the time of his service as an unpaid clerk in different situations there were no complaints against him; on the contrary, in Brünn his conduct is mentioned in the list of officials as blameless. 13. On the 11th January, at an examination of his house and person by the police, in consequence of a suspicion of his opening letters, the following articles were found in his possession:—

LIST.

1296 opened letters; 176 addresses of letters, besides a great number of new year's bills; 172 unmarked postage stamps taken off letters, 457 marked stamps, a box full of torn-off seals, a packet of paper from different offices, a packet of 14 sticks of sealing-wax, a small box with 4 pair of shirt buttons, 3 shirt buttons, 5 rings, and a small cross, 11 keys and 2 picklocks, 14 pieces of sealing-wax, 2 brass candlesticks, 5 tin cups, 1 coffee spoon and sugar basin, 2 knives and forks, 2 match holders, 1 purse, containing 1 silver gulden and 2 silver pieces, of the value of 50 Austrian kreutzers; 35 bank notes, 1 florin each; 5 per cent. State receipts for 100 florins, besides bonds; and lastly, a packet with documents belonging to the accused himself.

The prisoner acknowledged having stolen the greater proportion of the articles found. The idea of opening the letters in search of money had entered his mind shortly after his employment in the Post-Office; and he was incited thereto by the reproaches of his parents, who grudged the money they were compelled to send him. At the time of his imprisonment the prisoner had made no use of any of the stolen money. It was found in a box, and agreed pretty well with a list which he had kept of the letters and their money contents. He was in the habit of taking off stamped as well as unstamped postage stamps. The unstamped he sold, and the stamped he occasionally used for the transmission of letters from which he had removed the unmarked stamps, restamping them with the stamp of the day, rendering them so black as to attract the attention of the postman, which he supposed led to his detection. These confessions the prisoner made without any reserve. Five months after his incarceration the jail doctor was called, and found him in the following condition:—Eyes shut and unconscious, growling to himself, led up and down by a fellow-prisoner, at times starting from fright and muttering something about shooting, hands convulsed and the whole body tremulous, when the eyes were forcibly opened the pupils were found much dilated, while the iris was scarcely perceptible, and was quite insensible to light; eyelids closed convulsively, various other convulsive movements observed; colour very pale, hands cold and blue, pulse small and too rapid to be counted; was made to rise up and sit down at pleasure. The state of unconsciousness remained almost unbroken from the 22nd May till the 8th June. From the 10th June the prisoner appeared gradually to recover consciousness, but had no recollection of anything that occurred during his illness. He volunteered the information that an uncle of his had hanged himself, and a sister's child had, at the age of 20, gone out of his mind and set fire to a village. When asked why he mentioned these facts, he answered, because it had gone to his heart that he himself had become a fool. He mentioned subsequently that the attendants in the sick ward had told him he had lost his senses. He also said that, before his examinations, he had been perfectly well up in his subjects; but, when it came to the point, everything was forgotten. He spoke of several occasions on which he had fallen into a state of unconsciousness, sometimes not recovering from the effects of the fit for two or three days. He alluded without sorrow to his opening the letters, said he felt compelled to do so; that he had arranged all the letters according to their sizes. He gave a pitiable account of his voluntary frugality, from ten to eleven florins was the highest sum that he had ever spent in a month; he did not seem to know why he had lived so sparingly, said he had always plenty of money, that his father had often trusted him to collect accounts, and that he had always reckoned with him to the last farthing. He wept much when he recounted that his mother had fainted on receiving the news of his imprisonment.

### (B) OPINION.

1. It is evident, from the foregoing, that the prisoner had every desire to learn at school; and, in spite of the difficulty which he felt, applied himself with perseverance to prepare for the examinations; that even then he suffered from want of sleep, and that the power of thought at times left him quite suddenly, and that at least on three occasions he was seized with fits which left him unconscious and without the power of motion. 2. That both as a student and a clerk he had exercised a most uncalled-for frugality, and indulged a propensity to appropriate everything, even things without the slightest value, and to conceal and guard them as if they were treasures. Such an inclination at his age is quite abnormal, and betrays positive perversity. 3. It is further to be gathered that the prisoner had every desire to make progress in his calling; and we have positive testimony to his desire and endeavours, as fourteen days before his incarceration he had paid caution money to the amount of 400 florins. These objective facts place the subjective fact of his good intentions beyond a doubt. 4. The support which he received from his parents and his own store of ready money precluded any necessity for starving himself, and afforded him no temptation for the appropriation of what did not belong to him. 5. In spite of this, we find him living like a beggar, and secreting whatever he could lay his hands upon, at the very time that he was striving to obtain a situation with a good salary. 6, 7, 8. That the useless and aimless, as well as senseless, preservation of the opened letters, of the postage stamps and seals, the arrangement of the letters according to their size, the registering of the money found each day, the frank confession of what he had done, and the declaration that he had no idea why he did it, but felt himself compelled thereto, go far to prove that this opening of letters and purloining of various objects, proceeded, not from dishonesty, but was the consequence of a blind instinct, in fact, of the so-called "Kleptomania." 9. It is our opinion that this mental anomaly by no means comprehends the whole extent of the prisoner's derangement, which is the more probable, as some other members of the family, and his own illness in prison, gave token of insanity. 10. Further, we find in the present state of the prisoner symptoms which indicate material derangement of the brain, such as frequent headaches, continuous singing in the ears, blood-shot eyes, the occasional enlargement (unequally) of the pupils, the distortion of the mouth, otorrhœa, glandular swelling in the neck, irritation of the skin, continuously weak and rapid pulse. From these symptoms, and from the frequent bleedings at the nose, the acute inflammatory affections of the brain, accompanied by maniacal phenomena, we are of opinion that the prisoner suffers from plethora, or perhaps even tubercles in the brain. 11. We are of opinion that the prisoner, in consequence of derangement, in the form of kleptomania, is not responsible.

This opinion of Dr. Mauthner, physician to the Jail and Court of Justice, was fully confirmed by Dr. Emusy, town physician.

"As the crime had attracted considerable attention, the authorities felt themselves bound to submit our decision to the medical faculty at Vienna, who delivered an opinion entirely different from ours, holding that there were no sufficient grounds for excusing the prisoner on the plea of insanity, and that the crime itself was fully accounted for by the passion for money and accumulation."

Dr. Mauthner and Dr. Emusy adhered to their already expressed opinion, and, having continued their minute observation of the prisoner's conduct, brought forward additional confirmatory facts, such as that, besides the articles already mentioned, in the prisoner's possession was a box, containing a perfect *omnium gatherum*, but all arranged with scrupulous care, and the collection of which must have cost time as well as trouble. It was, moreover, found that the prisoner had not sold the unused stamps, as he had stated to be his intention; and it was proved that, during his incarceration, he was in the habit of stealing from his fellow-prisoners articles of no value, and, upon being remonstrated with, wept and promised amendment. Finally, these medical men (Drs. Mauthner and Emusy) arrived at the conclusion that the prisoner was much fitter for a lunatic asylum than for a jail.

Political disturbances broke out, which, for a time, caused the case to be thrown into abeyance; afterwards, however, Drs. Mauthner and Emusy were again called upon for their opinion. They reiterated their formerly expressed opinion as to the mental condition of the prisoner, and the inadvisability of retaining him in prison; and, as the medical faculty at Vienna coincided with them as to the prisoner's present mental condition, he was released from prison and sent home under the charge of a relative, who was commissioned to watch over the case and communicate from time to time with the authorities.

(*Casper's Vierteljahrsschrift*, Jan. 1862.)

2.—*On Insane Institutions and Insane Colonies.* By Dr. Flemming.

In place of giving brief and merely titular notices of the general contents of the last number of the *Allgemeine Zeitschrift für Psychiatrie,* which comprise articles upon the "Influence of the Moon upon Periodic Insanity," upon "Some forms of Insanity which might justly be committed to Lunatic Asylums," &c., we conceive that we may better meet the wishes and wants of our readers by presenting an expression of the opinions of one of the most destinguished alienists upon an all-engrossing topic in a Vidimus of Dr. Flemming's article, "Insane Institutions and Insane Colonies."

Dr. Flemming was commissioned by the meeting of German Psychologists at Eisenach to discuss the subject of Insane Colonies, and enters upon the subject with some hesitation, chiefly because he has not personally visited Gheel; he, however, proves that it is quite possible to arrive at accurate knowledge by other means. Dr. Flemming proposes to place the Asylum and Colony system face to face, and, comparing the differences existing in their accommodation, custody, and means of cure and care, to render the success in attaining the desired end appreciable. Dr. Flemming takes a survey of the case of the insane in the Middle Ages and refers to the unnoticed and unsuspected growth of the colony of Gheel. "Still, six years ago, disorder, blind routine, the spirit of speculation ruled in Gheel" (Bulckens). The state drew attention to Gheel, and, after inspection, improvements were begun.

Dr. Flemming mentions the exaggerated ideas of the colony held by some, and the controversy which has been going on, and says that the strenuous as well as the temperate advocates of Gheel have made concessions, by which they acknowledge the institution to be quite as necessary as the colony, and, in fact, indispensable for the completion of the latter system. The author goes on to examine the grounds of argument. The list of results given in Dr. Bulckens' reports for the last four years, is held by partisans to prove that the establishment of Gheel has done more for the cure of the insane than any other institution. The lists extend from 1855 to 1859, and include 505 patients.

LIST.

| | | |
|---|---|---|
| Recovered . . . . . . . . . | 100 or | 19·8 per cent. |
| Perceptibly better . . . . . . | 43 or | 8·5 per cent. |
| Retained from regard to the public safety . | 43 or | 8·5 per cent. |
| Ran away . . . . . . . . | 18 or | 3·5 per cent. |
| Dead . . . . . . . . . | 257 or | 50·8 per cent. |

Now, 19·8 per cent. of cures is no such very brilliant result that it should militate against the asylum statistics, which generally rise from 28 to 33 per cent.

One of those private asylums, so bitterly persecuted by the admirers of the colony system, and even accused of the most sordid selfishness, the Private Cure and Care Institution, Thouberg, Dr. Gunty, numbers during the 25 years of its existence:—

| | |
|---|---|
| Recovered . . | 57·2 per cent. |
| Better . . | 19·6 per cent. |
| Incurable . . | 8·2 per cent. |
| Dead . . . | 15·0 per cent. |

When Dr. Mundy, in order to show the benefits of Gheel as an establishment for cure, gives 96 cured out of 145, that is to say, a per centage of 66, we acknowledge the ingenuity of this method; but it is so little justifiable, that if we go a step further and hold that the real curables are only represented by the actually cured, whereby a per centage of 100 is arrived at, when the 4 recovered incurables are included it makes 104 per cent., a cipher which oversteps possibility itself.

Dr. Flemming is still far from disparaging the results of the method at Gheel; indeed they fill him with astonishment, when the probably large number of aged patients, and cases where the malady has been of long standing, and the simplicity of the treatment, are taken into consideration; but he asserts that it is not the number of cured which can place the colony system in the first rank of institutions for the insane, or give a pretext for setting aside all previously received systems. The author takes a survey of the classification of patients in Gheel from Dr. Bulckens' reports, and concludes that the arrangements are very similar to those in good asylums, only the distance of the individual portions is very materially greater, an arrangement, the propriety of which he should doubt, had Dr. Bulckens not expressly stated that no inconvenience has ever resulted.

Flemming speaks of the means of restraint used, the number of the medical staff, the medical prescriptions, the proposed infirmary, the alleged advantages of Gheel, which he thinks the patients in asylums also enjoy, except the asserted freedom, the propriety of which he does not seem to be quite sure of. The attendants at Gheel appear to suffer less from the unhealthy surrounding influences than the rational inhabitants of asylums. The author speaks of the patronage familial, and proves that in many cases it is quite inapplicable; but also allows its advantages, not only in the case of incurables, but also as a means of cure. Dr. Bulckens calls particular attention to one circumstance in which the colony system differs from that of the asylum, namely, that in the former the patients are not exposed to continual contact with their fellow-sufferers. The critic observes that if this be of great importance it should be more fully developed in Gheel, where two patients are generally found in each house, and the rules allow four.

Dr. Flemming considers the principal advantage of the separation of the insane to consist in their protection from the personal violence of each other, and attributes no such importance to it otherwise. In collecting the results of this discussion the following conclusions are arrived at:—

1. Colonies for the insane, which may be considered as institutions for care as well as for cure, cannot take the place of enclosed, or perhaps more properly secluded asylums, nor render them unnecessary, since the interests of the first demand the exclusion of a large number of the insane as unsuited for their privileges.

2. Colonies for the insane, even when brought to the greatest possible perfection, do not present any such advantages over the asylums as to render it possible or desirable that they should take their place; partly because (*a*) the colonies for the insane, in order to cure, must be arranged in a manner which, in reality, assimilates them to enclosed asylums; partly (*b*) the advantages which the colony offers for cure are only available for a small number of patients; partly (*c*) even these advantages are partially attained in well-regulated asylums, by means of suitable precautions, but still in greater measure than in colonies; partly (*d*) the disadvantages which in colonies for the insane proceed from difficulty of oversight and medical care, as well as from the want of scientific experience, outweigh the advantages, or at least, hold them in balance; partly (*e*) the results of treatment, according to the register of the colonies before us, by no means entitle them to the first place.

3. Colonies for the insane cannot, even as refuges for the incurable, supplant or, indeed, with the exception of the cheaper board, offer any advantages over enclosed asylums, which may not be approximated, or even exceeded in the latter. Even the advantage of a somewhat cheaper rate is compensated for by the necessity of erecting an infirmary for the completion of the colony.

4. In proportion as enclosed asylums fall short of colonies in any of these particulars do they obtain the advantage when we consider how much more practicable is the oversight, care, and arrangements for the physical weal of the inmates in the former than in the latter. These propositions suggest the question if it be not possible to combine the two systems, since the one cannot compensate for the other. A proposal to effect this end, by Dr. Bulckens (styled one of the most thoughtful among the advocates of insane colonies), is the union of the two systems by means of a special commission, whose business it shall be to classify the insane and to apportion them, according to their condition, to asylum-colonies, or enclosed asylums. Between the free and the enclosed asylum a continual interchange of patients would be kept up. The question of what arrangements are most suitable for the different forms of insanity is reserved to a further discussion.

The author brings forward against this proposition the great difficulties attending the transference of patients in most countries. Equal difficulties, the author says, beset the proposal of Dr. Mundy (one of the most learned of the advocates of the colonial system). A long description of Dr. Mundy's proposed combination system here follows. Dr. Flemming says, "he presents us with nothing more than the picture of one of our well-regulated institutions, only with its dominion extended over a larger area, where the care of the state is replaced by that of the family, the pensionat of the state by the pensionat of the family, and both placed under the same administration and medical direction." Here follows a long discussion, chiefly connected with the political economy of the system. Dr. Flemming deduces from what has been said the following conclusion:—

5. If it must be granted that colonies for the insane are the desideratum, and enclosed asylums merely subsidiary helps for certain cases, that is to say, (*a*) quiet imbeciles and idiots, (*b*) peaceful maniacs, (*c*) the moderately excited, (*d*) quiet melancholics, (*e*) convalescents; so, on the other hand, is the enclosed asylum the desideratum, and the colony merely the subsidiary for a much larger number of patients, that is, for (*a*) acute cases of excited and melancholy insanity, (*b*) the refractory and violent, (*c*) those of vicious inclinations and degraded habits, (*d*) indecent and dangerous to the public safety, (*e*) epileptic.

6. Colonies and enclosed asylums must consequently, when the one cannot suffice without the other, mutually supplement each other, and must co-operate or be combined.

7. The opportunity will seldom be found in Germany for the establishment of colonies to be united with such enclosed asylums as may still be built.

8. Just as little is it practicable in Germany to establish separate insane colonies in connexion with the already existing enclosed asylums.

9. Insane colonies to be united with already existing asylums would be more practicable, if apart from the unavoidable difficulties of formation and working, we expected any material lessening of the rate of board.

---

3.—*Report by* M. Jules Falret, *in the name of the Commission appointed by the Medico-Psychological Society of Paris to examine Gheel.* (*Annales Médico-Psychologiques*, Jan. 1862.)

This document, although expressing the opinions of the Commission composed of MM. Michea, Moreau, Mesnet, Trélat, and Baillarger, is not merely written exclusively by M. Jules Falret, but is, we have reason to believe, the result of his own observations alone.

The Reporter introduces the subject by a description of the territory and town, of the aspect of the population, sane and insane, and by the history, apocryphal and recent, of the colony; with which our readers must be familiar. He, however, introduces this novelty, that in past times, how remote is not stated, the adjudication of the residence of the lunatics was in some places conducted by auction; and at a board so low as twenty-four francs per annum. He describes the commune as being divided, medically, into four sections, each containing about two hundred lunatics; to each of which is attached a medical man, who, it would appear, is not an expert, but the communal, or, in our phraseology, the parish doctor. The central hospital is nearly completed—although designed for fifty patients only, it is capable of containing a hundred; and, according to M. Falret, will be at once filled, notwithstanding the predictions and wishes of the authorities, and the conditions which they have imposed as to admission. It would appear that lunatics are confided to the more affluent inhabitants, as well as to the peasants, and the charge is accepted as a privilege and a duty. The number of custodiers, moreover, appears to be increasing: in 1856 they amounted to 548, whereas in 1861 they reached 617; a change which must tend to diminish the number of patients in each house. The regulations that there shall be only one lunatic confided to each family, or if two, that they shall be of the same sex, is practically inoperative. The houses in which the insane are placed resemble those occupied by the rest of the population. Recently, buildings of particular form and dimensions, intended to secure the proper ventilation of the apartments assigned to the patients have been prescribed; but the alteration has resulted in small, unhealthy rooms, which, during winter, place the inmate in circumstances less favourable than what are met with in asylums. The diet of the host and the guest is precisely the same: which though sufficient for individuals in robust health, is deficient in animal food, and inappropriate to the invalid, the sedentary, and the sick. The dress is satisfactory, and the disappearance of the crowns of straw and all other extravagances is announced. While it is the interest and duty of each guardian to engage his charge in occupation, the "very great number" who are incapacitated by their physical or mental condition, or by their obstinacy, for active exertion is signalized. M. Falret found sixteen individuals restrained by chains or cinctures of iron at the time of his visit, and condemns unsparingly the frequent and prolonged recourse to such expedients; pointing out that at Gheel, as in asylums, they are applied chiefly to the same individuals. Escapes frequently occur, notwithstanding the physical and moral means which exist, or have been adopted, in order to prevent them; and the fines imposed when they take place. Not only at the first glance, but on minute examination of the colony, the impression received is truly favourable: not only do the insane appear domesticated with their guardians, but they generally speak in terms of praise and contentment as to the treatment which they receive, and make fewer complaints than what are heard from patients in asylums. The kindness and benevolence, the courage and sense of security displayed by the Flemings are placed in a pleasing light; but M. F. is, nevertheless, disinclined to regard them as belonging to an exceptional race, so endowed as to realize, in some sort, the golden age. There is an iron aspect of the subject; for there are curators who betray their trust, who enslave and inhumanly abuse their wards. The rarity of accidents, such as incendiarism, homicide, suicide, and prostitution, is commented upon; but the reporter insinuates that the patriotism, the community of feeling as well as of interests, are such among the inhabitants as to render the statistics obtained by the authorities in some degree untrustworthy. An impartial consideration of the actual position of Gheel leads M. F. to the conclusion that the inconveniences have been much exaggerated; and that, especially since recent reforms, the insane are in a better condition than what was previously conceived. He is not, however, one of the enthusiastic partisans of the institution, who sees in it an ideal paradise combining all the advantages desirable for the insane. He conceives that the importance of the two great and characteristic principles—the unrestrained personal freedom and the domestic life—which have given celebrity to the community, has been overstated and misunderstood. He argues against the applicability and the benefit of these as means of amelioration to all classes of lunatics.

The family life, like the asylum life, separates the patient from the injurious associations of home; but it deprives him of the resources of scientific treatment. The liberty of Gheel cannot be obtained elsewhere; but is it desirable? is it desired by the great majority of residents? is it real or illusory? M. Falret contends that a vast proportion of the insane, whether in seclusion or at Gheel, are utterly indifferent to a wider world than their own thoughts; that it is during lucid intervals, convalescence, and in certain dispositions that the repugnance to the restraint of seclusion is strong, and under circumstances when such restraint is useful and curative. Besides, though the removal of walls and bolts and bars is good in itself, there exist at Gheel most formidable, almost insurmountable barriers to escape; in the remoteness of the hamlet from other towns, in every inhabitant being a keeper, and deeply concerned in faithfully discharging his watch and ward, in the special supervision of suspected cases, in restraint mechanical and domiciliary, &c.

Admitting, however, the benefits which may be secured in certain cases from the arrangements at Gheel, M. Falret regards as counterbalancing every conceivable advantage, the negation of all active treatment and of all individual knowledge or management, on the part of the physicians. "We cannot forget all that has been written upon the general treatment of the insane." As to the moral and material well-being of the patients, it is concluded that even the chronic, the tranquil, and inoffensive, for whom Gheel is especially suited, are in a less satisfactory position, as to nourishment, clothes, and personal care, than in asylums; that the degraded, dirty, and those attacked by accidental diseases, are less cared for; and that the violent and epileptics are incontestably less happy and less suitably disposed of than in public establishments. He adds, as remediable objections, the presence of lunatics of different sexes and of too large a number in the same household, the inadequate classification, and the inability of the attendant to understand the language of his charge.

The colony, then, is neither so good nor so bad as contending parties have reported. It is not to be regarded as a system differing from that embodied in asylums; when united these are but the realization, more or less perfect, and by different means, of the principles which have been gradually developed for the last sixty years. Pinel inaugurated the truth that there should be accorded to the insane as much liberty as was possible, consistently with their own security and that of society. In M. Falret's opinion Gheel has more to gain by approaching the asylum system, than the asylum in approaching Gheel. Gheel is a Farm Asylum. He is convinced that a similar institution cannot be created. He quotes the cottage system in England; the plan of Roller, in Germany, of confiding chronic and inoffensive cases to the care of peasants in the neighbourhood of asylums; the almost abandoned farm connected with Bicêtre; the flourishing colony of Fitzjames [see last Number], as assimilations to the practice at Gheel. In colonization, under some form or other, he finds the best solution of the urgent questions as to the means of increasing the well-being of the insane; and of relieving public institutions of the surplus population of chronic cases, with which they are burdened, and he proposes four modes in which it may be accomplished:—

I. By placing patients who are greatly improved and inoffensive in their own families, under an official superintendence, exercised under the form of pecuniary aid and moral and medical advice.

II. By placing certain cases selected by the physician in the houses of peasants, or others, in the vicinity and under the superintendence of the medical officers of asylums.

III. By attempting the formation of a new Gheel, that is to say, a village of the insane, with a regular central organization, an earnest medical staff, and an hospital; with the express conditions, however, that there shall be received chronic cases only; and that there shall be excluded the excited, the dangerous, and those under treatment.

IV. By connecting with an asylum a farm, where patients would be received directly from, and, if necessary, sent back to the asylum, according to the opinion of the medical officer.

We may resume consideration of M. Falret's exhaustive report when we receive that which may be expected to emanate from the Committee of the Society of Medical Officers connected with Asylums. The spirit of the criticism appears liberal and impartial; but it is noteworthy that, whether M. Falret be infected with Anglophobia or not, he ignores altogether the examination of Gheel by English observers, and their opinions.

---

